# BARIATRIC SURGERY IN THE ELDERLY: RESULTS OF A MEAN FOLLOW-UP OF FIVE YEARS

**DOI:** 10.1590/S0102-6720201500S100006

**Published:** 2015-12

**Authors:** Denis PAJECKI, Marco Aurelio SANTO, Henrique Dametto Giroud JOAQUIM, Flavio MORITA, Daniel RICCIOPPO, Roberto de CLEVA, Ivan CECCONELLO

**Affiliations:** Bariatric and Metabolic Surgical Unit, Discipline of Digestive Surgery, Department of Gastroenterology, Hospital das Clínicas, Medical School, University of São Paulo, São Paulo, SP, Brazil

**Keywords:** Bariatric surgery/trends, Elderly

## Abstract

**Background:**

: Surgical treatment of obesity in the elderly, particularly over 65, remains controversial; it is explained by the increased surgical risk or the lack of data demonstrating its long-term benefit. Few studies have evaluated the clinical effects of bariatric surgery in this population.

**Aim:**

: To evaluate the results of surgical treatment of obesity in patients over 60 years, followed for an average period of five years.

**Method:**

: This was a retrospective study evaluating 46 patients, 60 years or older, who underwent surgical treatment of obesity, by conventional gastric bypass technique (laparotomy). The average age was 64 years (60-71), mean BMI of 49.6 kg/m^2^ (38-66), mean follow-up of 5.9 years; 91% of patients were hypertensive, 56% diabetics and 39% had dyslipidemia.

**Results:**

: The incidence of complications (major and minor) in patients under 65 years was 26% and over 65 years 37% (p=0.002). There were no deaths in the group with less than 65 years and there were two deaths (12.5%) over 65 years. The average loss of overweight over 65 years or less was 72% vs 68% (p=0.56). There was total control of the diabetes mellitus in 77% and partial in 23%, with no difference between groups. There was improvement in arterial hypertension in 56% of patients, also no difference between groups. The average LDL levels did not differ between the pre and postoperative (106 mg/dl to 102 mg/dl), an increase of HDL (56 mg/dl to 68 mg/dL) and reduced triglyceride levels (136 mg/dl to 109 mg/dl). There was no statistical difference in the variation of the cholesterol fractions and triglycerides between the groups. Two patients in the group with less than 65 years died in late follow-up, of brain tumor and pneumonia, three and five years after bariatric surgery, respectively.

**Conclusions:**

: Surgical morbidity and mortality were higher in patients over 65 years, and this group had the same benefits observed in patients lower 65 years for weight loss and comorbidities control.

## Abstract (pt)

**Racional:**

: O tratamento cirúrgico da obesidade em idosos, em particular nos indivíduos com mais de 65 anos, permanece controverso; seja pelo risco cirúrgico aumentado ou pela ausência de dados que demonstrem seu benefício em longo prazo.

**Objetivo:**

: Avaliar os resultados do tratamento cirúrgico em pacientes com mais de 60 anos, seguidos por um período médio de cinco anos.

**Método:**

: Estudo retrospectivo que avaliou 46 pacientes com 60 anos ou mais, submetidos ao bypass gástrico convencional (laparotomia). A idade média foi de 64 anos (60-71), IMC médio de 49,6 kg/m2 (38-66), tempo médio de seguimento de 5,9 anos. Pacientes eram hipertensos eram 91%, diabéticos 56% e 39% tinham dislipidemia.

**Resultados:**

: A incidência de complicações (maiores e menores) nos com menos de 65 anos foi de 26% e com mais de 65 anos de 37% (p=0,002). Não houve óbitos no grupo com menos de 65 anos e houve dois óbitos (12,5%) no com mais de 65. A perda média de excesso de peso nos pacientes com mais ou menos de 65 anos foi de 72% x 68% (p=0,56). Houve controle total do diabete melito em 77% dos pacientes e parcial em 23%, sem diferença entre os grupos com mais ou menos de 65 anos. Houve melhora da hipertensão arterial em 56% dos pacientes também sem diferença entre os grupos. Os níveis médios de LDL não variaram entre o pré e pós-operatório (106 mg/dl para 102 mg/dl), houve aumento do HDL (56 mg/dl para 68 mg/dl) e redução do triglicérides (136 mg/dl para 109 mg/dl). Não houve diferença estatística na variação das frações de colesterol e triglicerídeos entre os grupos. Dois pacientes do grupo com menos de 65 anos morreram no seguimento tardio por tumor cerebral e pneumonia, três e cinco anos após a cirurgia bariátrica, respectivamente.

**Conclusões:**

: A morbimortalidade cirúrgica nos pacientes com mais de 65 anos foi maior. Mas, os acima de 65 tiveram os mesmos benefícios observados nos com menos de 65 anos, em relação à perda de peso e controle de comorbidades.

## INTRODUCTION

In Brazil and in the so called "developing" countries, are considered elderly individuals aged 60 and over^25^. The incidence of obesity in this population ranges from 8% in men to 16% in women^23^. The goals of obesity treatment in this population are aimed at increasing survival without disability, reduction of musculoskeletal comorbidities and improvement in life quality^1^^,^ 10. However, there is a concern regarding excessive loss of muscle mass, loss of bone density, osteoporosis and increased risk of fracture, which can occur after massive weight loss^14^. In this context, the surgical treatment for cases of extreme obesity in this population remains controversial^12^^,^ 16.

The issue becomes relevant when population aging occurs, and a growing number of elderly patients with morbid obesity get in bariatric surgery services. In the United States 10% of bariatric procedures in academic centers occur in patients older 60 years. Many studies have been published showing similar surgical morbidity and mortality, comparing individuals with more or less 60^8.9^. However, some series, including authors experience, reported higher mortality in patients over 65 years^26^^,^ 27. Although the average weight loss seems to be lower in the elderly population, the control rates of co-morbidities are satisfactory^22^. Nevertheless, the results so far have not been sufficient to alter the NIH guidelines for bariatric surgery, which ultimately influence the behavior in the rest of the world.

Most studies published on the subject, whether prospective or retrospective, evaluating the results in relation to morbidity and mortality rates, weight loss and control of co-morbidities achieves follow-up not exceeding three years^2^^,^ 7 ^,^ 28. Longer-term follow-up are needed to better observe the benefits or potential harm to health of these patients, the massive weight loss, achieved through surgery could bring. 

In this sense, the objective of this study was to evaluate the results of surgical treatment of obesity in a number of patients over 60 years, followed for an average period of five years.

## METHODS

This is a retrospective study, which analyzed prospectively collected data from patients 60 years or older, who underwent surgical treatment of obesity in Bariatric and Metabolic Surgery Unit of the Department of the Digestive Tract Surgery of Hospital das Clinicas, University of São Paulo Medical School, from January 2004 to January 2012. Were included all patients who had completed 60 years at the time of operation. The surgical technique was the Roux-en-Y gastric bypass by laparotomy (conventional gastric bypass) being excluded patients undergoing other surgical method (adjustable gastric band or sleeve gastrectomy) or a revisional procedure. Data were collected by personal interview, telephone interview, and consultation to paper medical records and to electronic medical records, for the current test results. Were analyzed demographic data at the time of the operation, surgical morbidity, mortality rates and weight loss (percent loss of excess weight -% PEP), evolution of comorbidities (hypertension, type II diabetes, dyslipidemia), nutritional deficiencies ( iron, calcium, zinc, protein, vitamin D, vitamin B12) and late mortality. 

Statistical analysis compared weight loss, incidence of complications and mortality among patients over or under 65 at the time of operation. Therefore, the Student t test was used for the average weight loss and variation of results of laboratory tests, and chi-square for comparison of morbidity and mortality.

## RESULTS

Forty-six patients were analyzed who met the inclusion criteria. The mean follow-up was 5.9 years (71 months). Thirty patients were between 60 and 65 years and sixteen were over 65 years at the time of operation. The demographics of patients are shown in Table 1.

**TABLE 1:** - Patients over 60 years undergoing bariatric surgery from 2003 to 2012

**n**	**46**
Age (mean)	64 years (60-71)
BMI (mean)	49,63 kg/m^2^ (38-66)
gender	41 (89%) - women
Number of co-morbidities (mean)	3,7
Diabetes	26/46 (56.5%)
Hypertension	42/46 (91.3%)
Dislipidemia	18/46 (39.1%)
Follow-up (mean)	71 months (38-130)

Surgical complications and 90 days mortality are shown in Table 2.

**TABLE 2:** - Bariatric surgery in the elderly: complications and mortality

**Leaks**	**2 (4.3%)**
Bleeding	2 (4.3%)
Bowel obstruction	2(4.3%)
Wound infection	2 (4.3%)
Incisional hernia	5 (10.8%)
Deep venous thrombosis (DVT)	1 (2.2%)
Pulmonary embolism (TEP)	1 (2.2%)
Mortality	2 (4.3%)

The overall incidence of complications in patients analyzed between 60 and 65 years was 26.6% (8/30) and in over 65 years 37.5% (6/16) (p=0.002). There was no mortality in the group with less than 65 years and there were two cases (12.5%) in the group over 65 years. One death was due to pulmonary embolism and the other of sepsis secondary to infection of the surgical wound. The results regarding weight loss and comorbidity control are shown in Table 3.

**TABLE 3:** - Bariatric surgery in the elderly: weight loss and comorbidity control

%PEP*	**71,8%**
Partial control of diabetes **	6 (23%)
Full control of the diabetes ***	20 (77%)
Improvement of hypertension (medication reduction)	12/46 (26%)
Control of hypertension (without medication)	14/46 (30%)

* Ideal weight calculated from the BMI 25 kg/m^2^; ** glycated hemoglobin (A1c)> 6.5 g/dl with or without oral medication; *** glycated hemoglobin (A1c) ≤6.5 g/dl without oral medication

**TABLE 4:** - Variation of glycated hemoglobin, cholesterol fractions and triglycerides between pre and postoperative

	**Pre-operative (mean)**	**Postoperative (mean)**
Glycated hemoglobin -A1C (%)	6.73	5.7
LDL (mg/dl)	106	102
HDL (mg/dl)	56	68
Triglycerides (mg/dl)	136	109

The average weight loss in patients under 65 years was 68% and in aged 65 or more was 72% (p=0.56). There was no statistically significant difference in the control of diabetes, hypertension and dyslipidemia among patients with more or less 65 years.

The results of laboratory tests and related to nutritional deficiencies are shown inTable 5. There was no difference in the incidence of nutritional deficiencies among patients with more or less 65 years. The incidence of anemia in 46 patients was 6%.

**TABLE 5:** - Bariatric surgery in the elderly: laboratory tests related to nutritional deficiencies (average)

	**Pre**	**Post**
Hemoglobin (g/dl)	13.7	13.2
Ferritin (ng/ml)	162.7	121.8
Albumin (g/dl)	4.3	4,1
Total calcium (mg/dl)	9.1	9.0
Ionic calcium (mg/dl)	4.8	4.9
PTH (pg/ml)	70.7	78.8
Vitamin D (ng/ml)	21.7	22.9
Vitamin B12 ( (pg/ml)	489	744.2

There were two cases of late mortality (4.5%), three and five years after the operation, respectively. The first was the result of brain tumor (multiform glioblastoma) and the second of pneumonia, in a patient who had had intestinal obstruction in the early postoperative period. The two patients were less than 65 years when they were operated.

## DISCUSSION

Surgical treatment of obesity in the elderly is still controversial, especially for patients over 65 years. Although in the guidelines of the Brazilian Public Health System (SUS) age is not a limiting factor, the Brazilian National Health Agency does not consider mandatory coverage of this type of procedure for patients over 65 years, by the private health insurance companies^18^. For patients in this age group, the prevailing concept is that the risk/benefit of the procedure must be evaluated for each patient individually^24^.

In respect of the risk, it was shown that patients over 65 years have higher morbidity and mortality rates when compared to younger patients^30^. Nevertheless, bariatric surgery in this population was considered safe and with satisfactory results^19^.

This fact was observed in this study, which had higher morbidity (26.6% vs 37.5%) and mortality (0% vs 12.5%) in patients over 65 years. It should be noted that the incisional hernia (10.8%) and surgical wound infection (4.3%) occur in a higher incidence in bariatric operations by laparotomy. Although it is a global trend, the realization of laparoscopic bariatric procedures is not yet the reality for most patients treated by SUS. This may have been a bias in this study, since most of the publications that deal with bariatric surgery in the elderly, the procedures were done laparoscopically.

Two patients older than 65 years died as a result of early complications. One of them developed severe sepsis caused by wound infection. It is possible that this complication had not occurred after a laparoscopic procedure. The other patient had pulmonary embolism on the 7^th^ postoperative day. In a study that compared the incidence of this complication in patients over or under 60, no difference was observed^27^. But it is known that it increases progressively with age and is considered the leading cause of postoperative mortality. Comparative studies between conventional and laparoscopic gastric bypass, performed at the beginning of the "laparoscopic era" showed no difference between the two access, regarding the incidence of this complication^5^^,^ 20. More recently, Brolin et al.^4^ showed that age was predictor of mortality in patients undergoing conventional bypass, but not for those undergoing laparoscopic bypass. It is possible that the minimally invasive approach also contributes to reducing mortality in this population 17.

Regarding benefits, significant improvement of assessed comorbidities was shown, in proportion similar to that observed in the literature for patients over or under 60 and similar follow-up period^22^. The study is flawed in not evaluating the evolution of quality of life, which was due to the absence of pre-operative data. Nevertheless, it´s known that weight loss has great impact on improving the quality of life measured by questionnaires such as the SF-36^6^. In this sense, was considered quite satisfactory weight loss (% EWL=72%) and comparable to non-elderly population, which must have influenced positively in improving this aspect.

In the elderly there is a particular concern with the problem of loss of functionality^11^^,^ 13, which was shown in another publication, in different series^21^. The evolution of functionality in operated patients should be evaluated in a prospective study, with pre and postoperative data. Although the general impression is that patients improve their performance in activities such as standing, walking and climbing stairs, this is not always the case. The incidence of sarcopenia increases progressively from 65 years and a marked loss of muscle mass after surgery may lead to worsening of functionality^3^^,^ 31. An example was one of the patients who died in the late follow-up, by pneumonia. This patient had intestinal obstruction and was re-operated in emergency five days after the gastric bypass. Despite receiving nutritional therapy from the beginning, she developed malnutrition and muscle wasting. The patient developed walking difficulty and was restricted to wheelchair until her death, after five years. The incidence of nutritional complications in this study was small, but it´s not known if sleeve gastrectomy could have been better than the gastric bypass for these patients, considering these complications and loss of muscle mass. This question remains to be answered.

This study has evaluated the outcome of surgery in elderly patients with longer mean follow-up, and has addressed as well as the causes of late mortality of patients. The other patient who died in the late follow-up had a brain tumor (glioblastoma), not related to bariatric surgery. Research of this type of injury can hardly be performed systematically preoperatively in asymptomatic individuals. However, geriatric assessment routinely performed at our service includes the basic neurological exam and the application of cognitive tests. In the field of possible benefits, those with cognitive impairment or evidence of severe neurological disease are not considered candidates for surgical treatment.

## CONCLUSION

Surgical morbidity and mortality were higher in patients over 65 years, and this group had the same benefits observed in patients lower 65 years for weight loss and comorbidities control.
